# Deep Feature–Based Detection of Chiari Malformation Type I from Sagittal T2-Weighted MRI Using a Hybrid CNN–Machine Learning Framework

**DOI:** 10.3390/diagnostics16111583

**Published:** 2026-05-22

**Authors:** Zülküf Akdemir, Murat Canayaz

**Affiliations:** 1 Department of Radiology, Van Yuzuncu Yil University, 65000 Van, Türkiye; 2 Department of Computer Engineering, Van Yuzuncu Yil University, 65000 Van, Türkiye; mcanayaz@yyu.edu.tr

**Keywords:** Chiari malformation type 1, deep learning, ResNet-50, MobileNetV2, machine learning, MRI, logistic regression

## Abstract

**Objective:** Chiari Type I Malformation (CM1) is a structural abnormality of the hindbrain that can cause a range of neurological symptoms and often requires radiological confirmation using magnetic resonance imaging (MRI). The aim of this study was to develop and evaluate a deep feature-based machine learning framework for the automated detection of CM1 from sagittal MRI images. **Materials and Methods:** The cohort comprised 550 adults: 250 patients with CM1 (168 women, 82 men; age range, 18–65 years) and 300 healthy control participants (210 women, 90 men; age range, 18–65 years). A total of 764 T2-weighted sagittal MR images (384 CM1, 380 healthy) acquired from two different 1.5T MRI scanners (Siemens Magnetom Altea and Symphony) between 2020 and 2024 were retrospectively analyzed. Deep features were extracted using ResNet-50 and MobileNetV2 architectures and subsequently classified using Support Vector Machines (SVM), Logistic Regression (LR), Random Forest (RF), XGBoost, and voting-based ensemble models. Model performance was assessed through patient-level 5-fold cross-validation using accuracy, sensitivity, specificity, F1-score, PPV, NPV, and AUC metrics. Code and trained models are available from the corresponding author upon reasonable request; imaging data are not publicly available due to patient privacy and institutional restrictions. **Results:** Across patient-level five-fold cross-validation, models built on ResNet-50 deep features demonstrated extremely high and stable diagnostic performance. The final soft-voting ensemble classifier based on ResNet-50 achieved perfect mean performance, with accuracy, balanced accuracy, sensitivity, specificity, F1-score, and AUC all equal to 1.000 ± 0.000 across folds. Other ResNet-based classifiers also achieved near-perfect results. MobileNetV2-based models also demonstrated strong performance but showed slightly lower stability compared with ResNet-based models, with mean accuracies ranging from 0.984 to 0.993 and mean AUC values between 0.99947 and 0.99984 across classifiers. **Conclusions:** The proposed deep feature-based machine learning framework demonstrated excellent performance for the automated detection of Chiari Type I Malformation from sagittal MRI images. In particular, the ResNet-50–based soft-voting ensemble model achieved perfect classification performance in cross-validation testing, suggesting that deep feature representations combined with machine learning classifiers may serve as a promising computer-aided diagnostic tool for supporting radiological evaluation of CM1.

## 1. Workflow Implications

Chiari Malformation Type 1 remains a challenging condition to diagnose accurately, as traditional criteria based primarily on tonsillar herniation often fail to reflect the true clinical severity or guide surgical decision-making. Variability in radiologist interpretation, the subtlety of posterior fossa morphologic changes, and the absence of standardized quantitative biomarkers further complicate routine assessment. The near-perfect diagnostic performance achieved by the ResNet-based models in this study demonstrates a strong potential to enhance clinical workflows by providing objective, reproducible, and highly sensitive detection of CM1-related anatomical alterations on T2-weighted sagittal MRI. Such an automated system could support radiologists in distinguishing borderline or atypical cases, reduce misclassification in busy clinical settings, and improve triage for neurology and neurosurgery referrals. By maintaining its accuracy across different MRI scanners and image types, the proposed framework offers a practical path toward consistent CM1 evaluation across institutions. Ultimately, this approach may contribute to earlier diagnosis, more informed surgical planning, and improved overall patient management.

## 2. Introduction

Chiari Malformation Type 1 (CM1) is the most frequently encountered craniospinal junction anomaly within the group of Chiari malformations [1,2]. On magnetic resonance imaging (MRI), it is characterized by the descent of the cerebellar tonsils through the foramen magnum, exceeding the basion–opisthion line by 5 mm [3]. Previously reported to have a prevalence below 1%, CM1 is now estimated to occur in approximately 1–4% of the population due to the increasing use of MRI in brain and cervical scans in recent years [4]. While some patients exhibit no clinical symptoms and the condition is detected incidentally during radiological investigations, the most commonly reported symptoms are suboccipital headache, dizziness, and neck pain [5,6]. The exact etiopathogenesis of CM1 remains unclear. One proposed hypothesis involves insufficient development of the occipital somites and paraxial mesoderm following neural tube closure [4]. Since no laboratory biomarker has yet replaced cross-sectional neuroimaging in diagnosis, the importance of imaging has become even more pronounced.

In current clinical practice, CM1 assessment largely relies on measuring tonsillar herniation on sagittal MRI and the radiologist’s visual interpretation; however, this approach shows limited concordance with symptoms and surgical decision-making. Accordingly, various linear and volumetric posterior fossa analyses have been employed to diagnose CM1 and differentiate it from healthy individuals [7,8,9]. Nevertheless, the degree of herniation and other single-parameter assessments often do not fully reflect the clinical spectrum of CM1 [10,11,12]. Moreover, CM1 has been associated with a range of concurrent structural abnormalities, including hypoplasia and overcrowding of the posterior cranial fossa, platybasia, brainstem and midbrain configuration changes, and altered tonsillar/spinal dynamics [4,11,12,13]. Collectively, these considerations highlight the need for advanced imaging analysis approaches that can capture multivariate morphological patterns at the craniospinal junction rather than relying on isolated measurements. Recent AI-based studies have also highlighted the clinical heterogeneity of Chiari-related disorders. Gupta et al. used an AI clustering algorithm in a large cohort of pediatric patients with CM1 and syringomyelia and identified three distinct presenting phenotypes, supporting the value of data-driven approaches for characterizing complex Chiari-related patterns. Nevertheless, that work addressed phenotypic clustering rather than automated MRI-based detection of CM1, leaving a role for image-based diagnostic frameworks such as the one evaluated in the present study [14].

Artificial intelligence (AI) techniques are increasingly applied to support radiological image interpretation and may improve efficiency and consistency in pattern recognition tasks [15]. Convolutional Neural Networks (CNNs) can learn hierarchical image features relevant to pathology and have demonstrated high diagnostic performance in selected imaging applications. In this study, we aimed to evaluate deep learning–based image analysis models for CM1 detection on routine sagittal T2-weighted MRI by comparing ResNet-50- and MobileNetV2-derived deep feature representations combined with machine learning classifiers. We hypothesized that ResNet-50–derived features would provide higher discriminative performance than MobileNetV2-derived features. We envisioned this approach as an add-on decision-support tool to assist radiologists in detecting CM1 on routine sagittal T2-weighted MRI and to improve the consistency of CM1 assessment across readers.

## 3. Related Works

Previous artificial intelligence studies on Chiari Malformation Type I have mainly focused on MRI-based diagnostic classification, morphometric machine-learning approaches, and clinical outcome prediction. Lin et al. evaluated convolutional neural network performance for diagnosing Chiari Malformation Type I and investigated whether CNNs could distinguish craniovertebral junction morphology. Using T1-weighted sagittal MRI from 148 CMI patients and 205 healthy controls, they developed two models based on original cervical MRI and cropped posterior cranial fossa/craniovertebral junction images. Both models achieved high diagnostic performance, with validation accuracies of 100% and 97% and test accuracies of 97% and 96%, respectively. Their models also outperformed tonsillar herniation measurements in sensitivity, although limited interpretability was noted as an important limitation [16]. Similarly, Tanaka et al. assessed CNN-based models for diagnosing clinically significant CM1 in surgical candidates. In their cohort of 101 CM1 patients requiring surgery and 111 healthy individuals, VGG19 achieved 97.1% sensitivity, 97.4% specificity, and an AUC of 0.99, whereas ResNet-50 achieved 94.0% sensitivity, 94.4% specificity, and an AUC of 0.98 [17]. These studies demonstrate that deep learning models can achieve high diagnostic performance for CM1 on sagittal MRI; however, they mainly relied on end-to-end CNN-based classification strategies and did not systematically compare deep feature representations combined with multiple conventional machine-learning classifiers.

Other studies have emphasized morphometric and clinical variables rather than direct image-based deep feature extraction. Mesin et al. proposed a machine-learning approach to predict clinical deterioration and syringomyelia risk in Chiari I malformation using sagittal MRI-derived morphometric indices and patient-specific variables, including age and presenting symptoms. In 58 pediatric surgical patients, adverse outcomes occurred in 38% within one year, and the SVM-based algorithm improved accuracy from 62% with traditional methods to 71%; however, the relatively small and heterogeneous single-center cohort and short follow-up raised concerns regarding overfitting and generalizability [18]. Tetik et al. investigated whether posterior cranial fossa volume and odontoid-related anatomical measurements could complement tonsillar herniation assessment. Using T1-weighted MRI from 241 CM1 patients and five machine-learning algorithms, they analyzed 11 posterior cranial fossa-related morphometric parameters; after age and sex matching, 100 CM1 patients and 100 healthy controls were compared, and Random Forest achieved the highest accuracy of 100% [19]. These morphometric studies support the value of quantitative anatomical assessment in CM1, but they remain dependent on manually derived measurements and may therefore be affected by observer-related variability.

Recent AI-based studies have also extended beyond diagnosis toward postoperative or clinical outcome prediction. King et al. developed a deep learning–based method to predict symptom recurrence after surgery in CM1. In a retrospective cohort of 57 patients with preoperative and postoperative SF-12 data, ResNet-50 was used for feature extraction, and a CLAM-based weakly supervised multiple-instance learning approach was applied with five-fold cross-validation. The combined model incorporating MRI, SF-12 physical component score, and cerebellar ectopia achieved the best performance, with an AUC of 0.71 and an F1-score of 0.74 [20]. El-Hajj et al. used machine-learning models to predict 30-day readmission and reoperation after posterior fossa decompression in a large pediatric CM1 cohort of 7106 patients. Readmission and reoperation rates were 7.5% and 3.4%, respectively, and Random Forest achieved the highest performance for both readmission and reoperation prediction, with AUC values of 0.960 and 0.990, respectively [21]. Although these studies highlight the broader potential of AI in Chiari-related disorders, their primary focus was postoperative risk stratification or symptom recurrence rather than automated MRI-based diagnosis.

In contrast to the approaches summarized above, the present study evaluates automated detection of CM1 from routine sagittal T2-weighted MRI using a hybrid deep feature-based machine learning framework. Whereas previous studies primarily focused on T1-weighted CNN-based diagnosis, manual morphometric measurements, or postoperative outcome prediction, the present approach uses ResNet-50 and MobileNetV2 as frozen deep feature extractors and compares several downstream classifiers, including SVM, logistic regression, Random Forest, XGBoost, and voting-based ensemble models. In addition, both median and adjacent paramedian sagittal slices were included, and all performance estimates were obtained using patient-level five-fold cross-validation to reduce the risk of information leakage. Accordingly, the study evaluates whether deep feature representations extracted from routine T2-weighted sagittal MRI can provide stable and reproducible diagnostic support for CM1 detection.

## 4. Materials and Methods

### 4.1. Dataset, Labeling, and Preprocessing

This retrospective, single-center model-development study used patient-level five-fold cross-validation for internal testing. We developed and evaluated classification models intended as add-on diagnostic decision-support tools to assist radiologists and improve consistency across readers. Routine clinical cervical MRI examinations acquired on two 1.5-T scanners (Magnetom Altea and Symphony; Siemens Healthineers, Erlangen, Germany) were retrospectively obtained from the institutional PACS/clinical imaging archive between 2020 and 2024, including examinations from patients with CM1 and control individuals without CM1. No publicly available datasets or synthetic images were used. Potentially eligible participants were identified from institutional clinical records/registries and screened using predefined imaging and clinical criteria, with eligibility confirmed by radiologist review. Manual slice selection and label confirmation were performed on the institutional PACS/DICOM viewer (Mergentech PACS; Mergen Software Inc., Eskişehir, Türkiye; version 2.5.32-b622). DICOM images were de-identified by removing PHI from DICOM headers and replacing identifiers with study-specific codes, exported as lossless 8-bit grayscale PNG files, resized to 256 × 256 pixels using bilinear interpolation, and min–max normalized per image to the 0–1 range. No histogram equalization/CLAHE, z-score normalization, or manual window/level adjustments were applied. Single-channel images were replicated across three channels to match CNN backbone input requirements. Quality control was performed by an experienced radiologist, and nondiagnostic examinations, including motion artifacts, severe noise, or insufficient anatomic visibility, were excluded. All preprocessing and modeling steps were implemented in Python (Python Software Foundation, Beaverton, OR, USA) using TensorFlow v2.11.0 (Google LLC, Mountain View, CA, USA), Keras v2.11.0 (Google LLC, Mountain View, CA, USA), scikit-learn v1.2.2 (scikit-learn developers; fiscally sponsored by NumFOCUS, Austin, TX, USA), XGBoost v1.7.6 (DMLC/XGBoost developers, open-source software), NumPy v1.23.5 (NumPy developers; fiscally sponsored by NumFOCUS, Austin, TX, USA), pandas v1.5.3 (pandas developers; fiscally sponsored by NumFOCUS, Austin, TX, USA), and OpenCV v4.7.0 (Open Source Vision Foundation, Palo Alto, CA, USA).

The study included adults aged 18–65 years who underwent cervical MRI between 2020 and 2024 and were classified as CM1 or control using the prespecified imaging-and-symptom reference standard described below. Examinations were excluded for prespecified reasons, including prior craniovertebral/decompression surgery, relevant tumors or brainstem/spinal cord pathology, vascular lesions, and nondiagnostic image quality. The outcome variable was a binary patient-level diagnosis label (CM1 vs. control). A convenience sample of eligible examinations during the study period was selected. A total of 665 cervical MRI examinations performed between 2020 and 2024 were initially screened. After applying the prespecified eligibility criteria, 115 examinations were excluded due to prior craniovertebral or posterior fossa surgery (*n* = 33), craniovertebral junction or cerebellar tumor (*n* = 4), brainstem or spinal cord pathology (*n* = 27), intracranial aneurysm or vascular malformation (*n* = 3), and severe motion artifacts or inadequate anatomical visibility (*n* = 48). The final study cohort comprised 250 patients with Chiari Malformation Type I and 300 control participants (total, 550 individuals). For image-based modeling, these participants contributed 384 sagittal T2-weighted images in the Chiari malformation group and 380 images in the control group (total, 764 images).

CM1 status was assigned using a prespecified imaging-and-symptom reference standard and confirmed by manual radiologist review performed by a radiologist with 12 years of experience. Tonsillar descent was measured in millimeters on sagittal T2-weighted cervical MRI as the distance from the basion–opisthion line to the most caudal point of the cerebellar tonsil on the slice demonstrating the maximal descent, using the midline slice when visible or the adjacent paramedian slice with the best tonsillar visualization when necessary. A case was labeled CM1 if tonsillar descent was >5 mm and Chiari-related symptoms were documented in the clinical record at the time of imaging; controls were required not to meet the >5 mm criterion and to have no clinical diagnosis of CM1. Labels were not extracted from free-text imaging reports or electronic health records using natural language processing. Because labeling was performed by a single reader, interreader agreement, adjudication, and discrepancy resolution procedures were not applicable. This reference standard reflects commonly used clinical diagnostic criteria and can be applied consistently to routine cervical MRI examinations, aligning with the intended use of the algorithm; however, label noise may persist because the 5 mm threshold does not fully capture clinical severity, symptoms may be nonspecific, and tonsillar descent measurements are subject to technique- and observer-related variability, particularly in borderline cases. Interrater variability was not assessed because a single radiologist performed reference standard labeling. Intrarater variability was not measured. Variability was mitigated using a prespecified protocol, standardized slice-selection rules, and exclusion of nondiagnostic examinations.

Median and adjacent paramedian sagittal T2-weighted slices in which tonsillar herniation could be identified were selected manually using predefined criteria, prioritizing images that best demonstrated tonsillar herniation and satisfied the study’s herniation criterion. Selection prioritized the midline slice and, when the tonsils were better visualized off-midline, the adjacent paramedian slice(s). One or more slices per patient were selected based on which slice best demonstrated tonsillar herniation. The patient-level label was propagated to all selected slices used for model development. Predictor variables consisted of the selected sagittal T2-weighted slices after resizing to 256 × 256 pixels; no nonimaging clinical variables were used as model inputs. Age, sex, and scanner type were recorded for descriptive and subgroup reporting only. No imputation was performed; examinations with incomplete or nondiagnostic sagittal T2-weighted inputs required for analysis were excluded during case selection.

#### Data Partitioning and Internal Validation

Model development used patient-level five-fold cross-validation. In each iteration, approximately 80% of patients, corresponding to four folds, were used for training, and the remaining 20%, corresponding to one fold, served as a held-out internal testing fold. To prevent information leakage, partitions were disjoint at the patient level, such that all sagittal slices from the same individual were assigned to the same fold. Fold assignment was performed to maintain a comparable case–control distribution across iterations, and a fixed random seed was used (seed = 42) for reproducibility. Because classifiers were trained using prespecified/default hyperparameters, no separate tuning/optimization partition was created; performance was summarized across the five held-out internal testing folds.

No external testing cohort was available; therefore, all reported metrics represent internal testing on held-out folds rather than evaluation on an independent population. The same inclusion criteria, labeling protocol, and preprocessing pipeline were applied uniformly across folds. Images from both 1.5-T scanners were represented within each fold to avoid systematic domain separation; thus, performance reflects internal generalization within single-institution acquisition variability. The absence of external testing is acknowledged as a limitation and motivates future multi-center evaluation.

Participant characteristics across data partitions were assessed to ensure fair and spectrum-balanced internal evaluation. The final dataset comprised 250 CM-I patients and 300 control participants, with age and sex distributions recorded for all individuals: the CM-I group included 168 females (67.2%) and 82 males (32.8%), and the control group included 210 females (70%) and 90 males (30%). Demographic characteristics were examined separately for each held-out test fold in the five-fold patient-level cross-validation framework. In each iteration, approximately 80% of patients were assigned to the training folds and 20% to the test fold, with all slices from an individual allocated to the same partition. For each test fold, we verified that the number of CM-I and control participants, age distribution, sex distribution, and scanner type (Magnetom Altea vs. Magnetom Symphony) were comparable across folds, confirming similar demographic and acquisition characteristics between folds. Model performance metrics were reported as the mean ± standard deviation across the five held-out test folds, reflecting variability attributable to changes in patient composition across partitions.

Model performance was evaluated exclusively on the held-out test fold in each iteration of the patient-level five-fold cross-validation framework. All metrics reported in this study therefore reflect performance on five independent test partitions, each containing approximately 20% of patients, with no patient overlap between training and test data. For the final model, defined a priori as the classifier achieving the highest mean AUC across folds, diagnostic performance was summarized as the mean ± standard deviation of accuracy, sensitivity, specificity, F1-score, and AUC across the five held-out test folds.

### 4.2. Cervical MRI Examination

**Figure 1:**
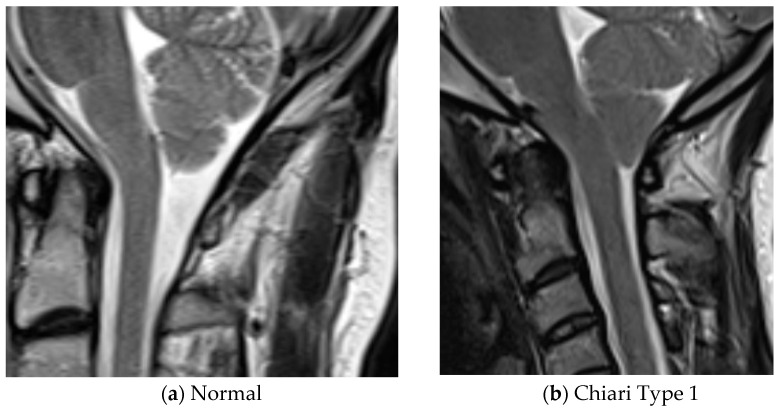
Dataset Samples.

Cervical MRI examinations were acquired on two 1.5-T systems (Magnetom Altea and Magnetom Symphony; Siemens Healthineers, Erlangen, Germany). Sagittal T2-weighted imaging parameters were TR, 3520 ms; TE, 89 ms; matrix, 240 × 320; FOV, 20 × 18 cm; section thickness, 3 mm. The dataset sampling is shown in Figure 1.

### 4.3. Deep Feature Extraction

To extract meaningful features from the images, ResNet-50 and MobileNetV2 deep learning models were used. Transfer learning was applied using pretrained versions of these models, and only feature extraction was performed. The fully connected (FC) layers of these models were removed, and feature vectors were extracted from the final convolutional layers for each image. The CNN backbones were initialized with pretrained ImageNet weights, and all convolutional layers were kept frozen throughout the study. A shallow classification head composed of fully connected layers was trained on top of the frozen convolutional base, allowing the model to adapt high-level representations to the specific characteristics of the dataset while preserving the generalization capability of pretrained features. No fine-tuning of convolutional layers was performed. This hybrid design was preferred to balance feature representation power and interpretability. Using frozen CNN feature extractors reduces the risk of overfitting in relatively small datasets and allows downstream classifiers to operate on stable and transferable representations. This approach ensures that the learned feature representations are largely driven by pretrained knowledge rather than dataset-specific overfitting.

The dimensionality of the extracted deep features differed between the two CNN architectures. Specifically, the ResNet-50 backbone produced 1024-dimensional feature vectors per image, resulting in a feature matrix of shape (764, 1024), whereas MobileNetV2 generated 1280-dimensional feature vectors, corresponding to a feature matrix of shape (764, 1280). To reduce redundancy and improve generalization, feature dimensionality was reduced using Random Forest–based feature importance, and the 50 most informative features were retained for downstream classification. This dimensionality reduction step was also intended to mitigate overfitting, given the relatively limited sample size compared with the high-dimensional feature space. Importantly, feature selection was performed strictly within the training folds of each cross-validation iteration, ensuring that no information from the held-out test data was used, thereby preventing data leakage. The difference in feature dimensionality between the two architectures reflects their internal design, with MobileNetV2 producing higher-dimensional representations due to its depthwise separable convolution structure. However, the subsequent feature selection step ensured a consistent and compact feature representation across models, enabling a fair comparison of downstream classifiers. Classical machine-learning classifiers, including SVM, Logistic Regression, Random Forest, and XGBoost, were trained using their default scikit-learn or XGBoost hyperparameters, with reproducibility ensured through fixed random_state values where applicable.

### 4.4. Machine Learning Models and Classification

Before model training, all performance estimation procedures were conducted using patient-level five-fold cross-validation. The dataset was randomly partitioned into five mutually exclusive folds of approximately equal size, and all slices from the same patient were assigned to the same fold to prevent information leakage. In each iteration, four folds (≈80%) were used for training, while the remaining one fold (≈20%) served as the held-out test fold. Because all classifiers were trained with predefined default hyperparameters, no separate tuning or validation subset was created. All performance metrics reported in the Section 5 therefore represent the mean performance across the five held-out test folds.

Features extracted from deep learning models were classified using conventional machine learning algorithms. The classifiers used are listed below:

- Logistic Regression: Creates linear decision boundaries and generally demonstrates strong generalization performance with high-dimensional feature spaces.
- Support Vector Machines (SVM): A powerful classifier that identifies the optimal separating hyperplane.
- Random Forest: An ensemble of decision trees known for its robustness against overfitting.
- XGBoost: A gradient-boosting-based ensemble algorithm that demonstrated strong stability with high accuracy and low variance across folds.

Multiple pipelines were evaluated by combining two deep feature extractors (ResNet-50 and MobileNetV2) with six downstream classifiers (logistic regression, SVM with an RBF kernel, random forest, XGBoost, and soft/hard voting ensembles). For each pipeline, performance was estimated using the five-fold patient-level cross-validation described above. Because hard voting outputs only discrete class labels (i.e., no continuous decision scores or probabilities), AUC-ROC (and DeLong comparisons) were not computed for this model; instead, it was assessed using threshold-based metrics (e.g., F1-score and balanced accuracy). Mean AUC-ROC was chosen as the primary selection criterion for models providing continuous scores/probabilities because it is threshold-independent and reflects overall discriminative ability, which aligns with the clinical objective of distinguishing CM-I from controls. In the event of ties or near-equivalent mean AUC-ROC values (absolute difference < 0.005), F1-score and balanced accuracy were used as secondary criteria.

Based on this predefined hierarchy, the ResNet-50 feature extractor combined with the soft-voting classifier achieved the highest mean AUC across all folds and was therefore designated as the final model for reporting and interpretation. No separate tuning or external validation set was used for final model selection, and no secondary “alternative final model” was retained, as no other pipeline provided a clinically meaningful advantage in computational efficiency or interpretability.

No data augmentation was applied, as the objective was to evaluate the intrinsic diagnostic value of native anatomical information without introducing synthetic spatial transformations. Deep features were obtained using pretrained ResNet-50 and MobileNetV2 backbones initialized with ImageNet weights. The fully connected layers were removed, and feature vectors were extracted from the final convolutional block outputs. All convolutional parameters were kept frozen, and no fine-tuning or training of randomly initialized layers was performed, ensuring deterministic and reproducible feature extraction.

For each cross-validation fold, the extracted feature vectors were used to train logistic regression, RBF-SVM, random forest, XGBoost, and soft/hard voting classifiers. No hyperparameter tuning was conducted; all classifiers used their default settings (Table 1). Logistic regression used max_iter = 100, tol = 1 × 10^−4^; RBF-SVM used tol = 1 × 10^−3^ with max_iter = −1; random forest used n_estimators = 100; and XGBoost used n_estimators = 100, objective = “binary:logistic”, and eval_metric = “logloss”, consistent with the need for probabilistic outputs in diagnostic decision-making. Across the 5-fold cross-validation, a total of 5 × 2 × 6 = 60 models were trained. The a priori criterion for identifying the best approach was the highest mean AUC-ROC across the held-out folds, consistent with the clinical goal of maximizing discrimination between CM-I and control subjects.

Ethical approval for this retrospective study was obtained from the Non-Interventional Clinical Research Ethics Committee (decision no. 2025/02-19). Because of the retrospective nature of the analysis, the requirement for written informed consent was waived.

#### 4.4.1. Statistical Analysis

Model performance was primarily assessed using the area under the receiver operating characteristic curve (AUC–ROC). AUC was selected as the primary metric because it evaluates discrimination across all possible decision thresholds and is therefore well-suited to the clinical objective of distinguishing CM-I from controls without relying on a fixed operating point. Secondary metrics included accuracy, balanced accuracy, sensitivity (recall), specificity, positive predictive value (precision), negative predictive value, and F1-score, all derived from the confusion matrix of the held-out test folds. Sensitivity and specificity were reported to reflect the clinically relevant trade-off between missed CM-I cases (false negatives) and unnecessary referrals (false positives), which are central concerns in the diagnostic workflow of CM-I. For models producing probabilistic outputs, AUC values were statistically compared using DeLong’s test, allowing assessment of whether observed differences in discrimination were statistically meaningful.

Model performance across the 5-fold cross-validation was summarized as mean ± standard deviation (SD) for all metrics, including accuracy, balanced accuracy, sensitivity, specificity, precision, negative predictive value, F1-score, and AUC. To quantify uncertainty in discrimination, 95% confidence intervals (CIs) for AUC were estimated using a patient-level, nonparametric bootstrap (2000 resamples) applied to the out-of-fold predicted probabilities, ensuring that each patient contributed only one prediction to the analysis. Pairwise differences in AUC between models were assessed using DeLong’s test on the same paired out-of-fold predictions (two-sided α = 0.05). Differences in paired binary classification decisions at a fixed operating threshold (0.5) were evaluated using McNemar’s test; when the discordant cell count (b + c) equaled zero, McNemar testing was considered not applicable. All statistical analyses were performed in Python using SciPy (v1.10), statsmodels (v0.14), scikit-learn (v1.2.2), and an open-source DeLong implementation compatible with NumPy (v1.23.5).

To comprehensively evaluate the diagnostic performance of the models developed in this study, we applied a multilayered statistical analysis framework. Because this was a retrospective study, the sample size was determined by the number of eligible examinations available during the study period. To contextualize adequacy for the primary endpoint (between-model AUC difference), we evaluated the expected precision and detectable effect size. With 250 CM1 cases and 300 control participants, the standard error of an AUC in the range observed for the MobileNet-based models (≈0.85) is approximately 0.017 (95% CI half-width ≈ 0.033). Under a conservative assumption of independent AUC estimates, this sample size provides approximately 83% power to detect an AUC difference of 0.07 and approximately 91% power to detect a difference of 0.08 at a 2-sided α of 0.05; because DeLong’s test compares correlated ROC curves on the same cases, the actual power is expected to be higher. For near-ceiling performance (AUC approaching 1.00, as observed for the ResNet-based models), uncertainty estimates and between-model testing may be less informative because misclassifications are rare and the effective information for discrimination is limited; therefore, confidence intervals and comparative *p* values should be interpreted in this context.

As noted above, in some instances the two models classified all cases identically under the default decision threshold of 0.5, and the McNemar test prerequisite (b + c > 0) was not met. To overcome this limitation and to examine how sensitive the models’ clinical decision-making behavior is to changes in the decision threshold, we performed a detailed threshold analysis across a range of 0.1 to 0.9. This analysis demonstrated how sensitivity, specificity, and accuracy varied across different decision thresholds; it also showed that the models diverged substantially at certain thresholds, rendering McNemar’s test applicable at those operating points. Thus, the models were compared not only in terms of AUC performance, but also in terms of how they would be expected to behave in clinical practice across varying levels of risk tolerance.

To assess which anatomical regions most influenced model predictions, we generated case-level visual explanation maps using an occlusion sensitivity approach, which is compatible with our feature-extraction + classical machine-learning pipeline. For each test image, a square patch (24 × 24 pixels) was systematically masked across the sagittal T2-weighted image using an 8-pixel stride. At each mask position, the full inference pipeline—including CNN feature extraction and classifier prediction—was re-run, and the resulting change in the predicted probability of CM-I (Δp) was recorded. The Δp values were assembled into a saliency map, upsampled to full resolution, and overlaid on the original MRI to visualize regions with the strongest influence on the model’s decision.

The following metrics were used to compare model performance:

- Accuracy: Indicates the proportion of correct classifications made by the model.
- Precision: A metric that minimizes false positives, measuring how accurate the model’s positive predictions are.
- Recall: Reflects the model’s ability to detect positive classes.
- F1 Score: The harmonic mean of precision and recall; important for imbalanced datasets.

The models were trained using default parameter settings. These values are provided in Table 1 below. All machine learning models were trained using default hyperparameters to ensure reproducibility and to avoid overfitting due to excessive parameter tuning. This approach provides a fair baseline comparison across models.

#### 4.4.2. Enhancing Results with Voting Classifier

An ensemble model was constructed using three base classifiers: SVM (RBF kernel), Random Forest, and XGBoost. In the soft voting configuration, each base classifier produced a probability estimate for each class, and these probabilities were combined using equal weights (weights = None). For each sample, the final predicted class corresponded to the class with the highest averaged probability across the three models. In the hard voting configuration, the final prediction was determined by majority vote over the discrete class labels output by the base classifiers. Because hard voting does not generate calibrated probability estimates, AUC-ROC and DeLong comparisons were not computed for this model.

The VotingClassifier was trained independently within each training fold of the 5-fold cross-validation procedure, and the corresponding held-out fold was used exclusively for performance evaluation, ensuring that ensemble predictions for each case were based solely on models that had not seen that patient during training.

### 4.5. Models

#### 4.5.1. ResNet

Residual Network (ResNet) is an architecture that introduced a significant innovation in the field of deep learning, particularly in image recognition and computer vision applications. The most notable feature of this architecture is its effective resolution of vanishing gradients and degradation problems encountered in training very deep networks. While traditional deep neural networks struggle with declining performance as the number of layers increases, ResNet overcomes these challenges through the concept of residual learning. In this approach, each layer transmits the input to the next layer while also adding a residual, or difference, with the input. This method facilitates training and enables the successful training of deep models.

The basic structure of ResNet consists of residual blocks. These blocks use an identity mapping mechanism that passes the input directly to the output. Mathematically, this is expressed as a residual block, where the function to be learned represents the input, and if this function is close to zero, the output becomes the input itself. This feature allows the network to deepen without performance loss. Furthermore, residual connections enable gradients to be directly transferred to subsequent layers, thus minimizing the vanishing gradient issue. Thanks to these properties, ResNet allows for the successful training of models with over 100 layers and is considered a major milestone in deep learning. This model, with its deep architecture and filters learned at a large scale, offers highly discriminative features.

#### 4.5.2. MobileNet

MobileNet is a lightweight and efficient convolutional neural network (CNN) architecture optimized for deep learning-based image recognition and classification tasks. Developed by Google, this open-sourced model was specifically designed to run on mobile and embedded devices. MobileNet significantly reduces both computational cost and model size by replacing traditional convolutions with depthwise separable convolutions. As a result, it provides high accuracy even on low-power devices and is widely used in applications such as object recognition, facial recognition, and augmented reality. Moreover, improved versions like MobileNetV2 and MobileNetV3 have been developed to suit different use cases.

### 4.6. Method

**Figure 2:**
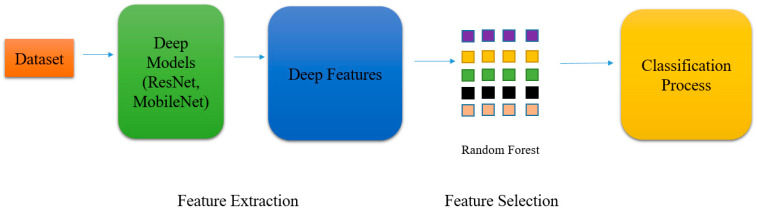
The proposed method. The colored blocks schematically represent different components/groups of the extracted deep features.

In our study, deep features were extracted using ResNet-50 and MobileNetV2 deep learning models. Based on the extracted features, 50 features were selected using the Random Forest method. These features were first classified by selecting the best-performing classifier among the five classifiers using the voting classifier. In the voting classifier, the k-fold value was set to 5 to ensure the reliability of the results. Subsequently, the individual performances of the classifiers were also evaluated with the k-fold value set to 5. For the two classifiers that achieved high accuracy, their performances were further demonstrated by setting the test size ratio to 0.3. The proposed method is illustrated in Figure 2.

## 5. Experimental Results

In this study, we comprehensively compared the performance of multiple machine learning classifiers built on deep feature extraction derived from ResNet- and MobileNet-based architectures. The results indicate that the models differed substantially with respect to diagnostic accuracy and discriminative ability. The comparative results for the voting classifier are presented in Table 2.

The table shows that ResNet-based feature representations achieved perfect classification performance across all evaluated classifiers (SVM, RF, LR, XGB, and voting methods) on the hold-out test set, with Accuracy, Balanced Accuracy, Sensitivity, Specificity, and F1-score all equal to 1.000. The equality between Accuracy and Balanced Accuracy indicates that performance was not influenced by class distribution and that both classes were classified without error. For the MobileNet-based features, accuracy ranged between 0.967 and 0.983. Specificity remained 1.000 across all classifiers, indicating the absence of false positives. In contrast, Sensitivity values (0.933–0.967) suggest that the observed errors were exclusively false negatives. The consistency between PPV values (1.000) and slightly lower NPV values (0.938–0.968) further confirms this confusion matrix structure. Overall, the reported metrics are mathematically coherent and internally consistent, with no contradiction among performance indicators.

All models achieved an AUC of 1.000 (where applicable), indicating near-perfect class separability in the learned feature space. The small performance gap observed in Accuracy between ResNet and MobileNet is therefore likely attributable to threshold-based classification differences rather than limitations in ranking capability. Furthermore, the consistent performance hierarchy across all classical classifiers suggests that the primary determinant of performance is the quality of the extracted deep feature representation rather than the downstream classifier itself. The equality of Accuracy and Balanced Accuracy values, along with the stable metric relationships across models, supports the statistical coherence of the results and indicates the absence of metric inflation or imbalance-driven bias.

Table 3 presents the results of six classification algorithms, including the voting classifier.

As shown in the table, no statistically significant difference was observed between the ResNet- and MobileNet-based models. The McNemar test results (*p* > 0.05 for all classifiers) indicate that the two architectures produce statistically comparable classification decisions at the sample level. Furthermore, both architectures achieved an AUC value of 1.000, demonstrating complete class separability within the feature space and equivalent discriminative capacity. Given that the AUC values are identical, the DeLong test naturally yielded non-significant results (*p* = 1), which is statistically expected and methodologically consistent.

Since the hard voting strategy does not generate probability estimates, AUC and DeLong comparisons were not applicable and were therefore appropriately reported as N/A. Overall, the McNemar and DeLong analyses provide mutually consistent evidence, with no statistical contradiction between decision-based and probability-based performance measures. These findings indicate that both architectures exhibit comparable generalization performance on the test set and that the reported results are statistically coherent and methodologically sound.

In addition to the results presented in Table 3, as noted in the Section 4.4.1, model performance was also evaluated across a range of decision thresholds. These results are provided in Table 4.

A threshold sensitivity analysis was conducted to evaluate the stability of the ResNet- and MobileNet-based models under varying decision thresholds. The ResNet-based model achieved 100% accuracy across all evaluated threshold levels (Accuracy = 1.00). In contrast, the MobileNet-based model exhibited a limited number of misclassifications as the decision threshold increased.

The McNemar test was performed using a paired 2 × 2 contingency table constructed as shown in Table 5.

Where

- a represents the number of samples correctly classified by both models.
- b represents the number of samples correctly classified by ResNet but misclassified by MobileNet.
- c represents the number of samples misclassified by ResNet but correctly classified by MobileNet.
- d represents the number of samples misclassified by both models.

Across all threshold values:

- c = 0, indicating that there were no instances where MobileNet outperformed ResNet.
- The a cell ranged between 56 and 60, demonstrating that both models correctly classified the majority of samples.
- The d cell was consistently 0, meaning there were no instances where both models simultaneously misclassified the same sample.

For example:

- At threshold = 0.5: a = 58, b = 2, c = 0, d = 0
- At threshold = 0.9: a = 56, b = 4, c = 0, d = 0

Since the McNemar test is based solely on the discordant pairs (b and c), and given that c = 0 while b remained small (b ≤ 4), no statistically significant difference was detected between the two models across threshold levels (*p* > 0.05).

These findings indicate that both models largely agreed on correctly classified samples and that no systematic divergence in performance was observed. Nevertheless, the ResNet-based model demonstrated greater stability under threshold variation, maintaining perfect classification performance across all evaluated decision levels.

Table 6 compares the mean k-fold performance of five machine learning algorithms (SVM, RF, LR, XGB, and Voting) trained using MobileNet- and ResNet-based feature extraction. The results clearly demonstrate the difference in discriminative performance between the two architectures.

When the five-fold cross-validation results are examined, it is observed that ResNet-based deep features provide extremely high and stable performance across all machine learning algorithms. In particular, for the SVM, RF, LR, and Voting Soft models, the mean values of all performance metrics (Accuracy, Balanced Accuracy, Sensitivity, Specificity, F1, and AUC) were found to be 1.000, with standard deviations close to zero. This indicates that the model performance is highly consistent across folds and that the variance is negligible.

For MobileNet-based features, although the performance values are also very high, a small but measurable variance is observed compared to ResNet. Notably, in the XGBoost model, the standard deviation values are relatively higher than in the other models (Accuracy std ≈ 0.009). Nevertheless, within the MobileNet architecture, AUC values are at the level of 0.999, indicating very strong discriminative capability.

In both architectures, AUC values ranging between 0.997 and 1.000 demonstrate that the dataset possesses strong class separability. The lower variance and higher average performance of the ResNet-based models suggest that this architecture provides a more stable feature representation.

However, since the number of folds is limited to five, statistical inferences are based primarily on variance analysis, and overgeneralization should be avoided. In this context, the results indicate that ResNet shows a consistent superiority over MobileNet, while both architectures achieve high-performance classification.

In addition, baseline models using DummyClassifier(scikit-learn developers; fiscally sponsored by NumFOCUS, Austin, TX, USA) were evaluated to assess chance-level performance. These models yielded identical results across both ResNet and MobileNet feature representations, which is expected because the DummyClassifier does not utilize input features and instead relies solely on the class distribution in the training data. The baseline accuracy remained close to chance level, confirming that the classification task is non-trivial.

The substantial performance gap between baseline and proposed models indicates that the observed results are attributable to the discriminative power of the extracted deep features rather than dataset simplicity. For the DummyClassifier, sensitivity and specificity values are inherently dependent on the class distribution, since the model always predicts the majority class and does not incorporate feature-based learning. Therefore, these metrics should be interpreted cautiously and are reported primarily to illustrate baseline behavior.

**Figure 3:**
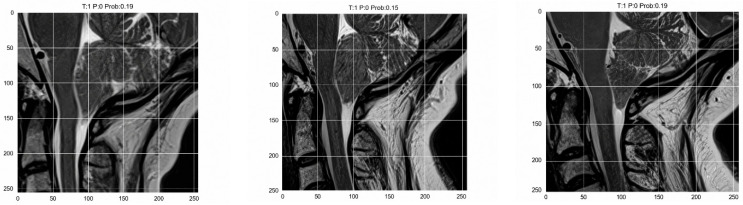
Representative failure cases from the MobileNet-based model. Misclassified samples are presented along with their predicted probabilities. These cases are characterized by low-confidence predictions, indicating that the model expresses uncertainty when encountering challenging or ambiguous anatomical patterns.

To further investigate model behavior, failure-case and borderline-case analyses were conducted. As shown in Figure 3, the ResNet-based model did not produce any misclassifications, while the MobileNet-based model yielded a small number of errors (*n* = 3). Visual inspection of these failure cases indicates that misclassifications are associated with low-confidence predictions.

**Figure 4:**
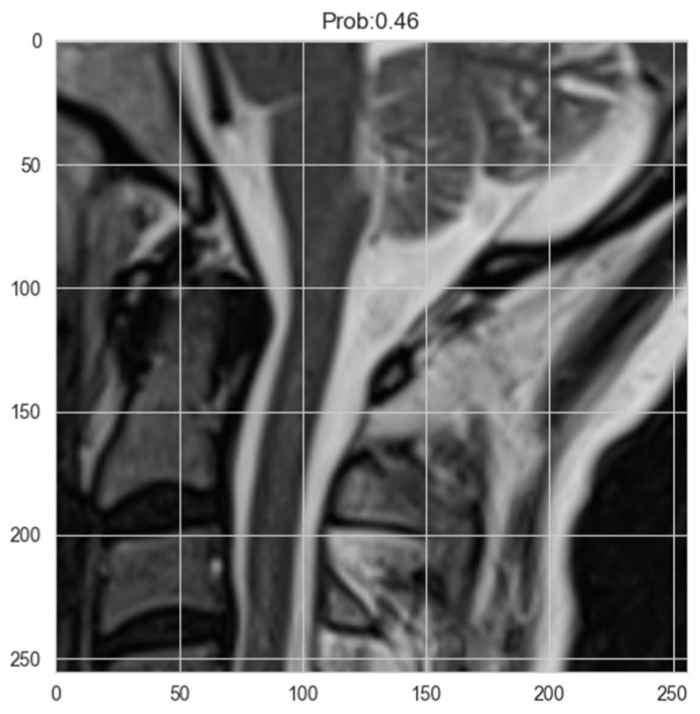
Borderline cases with prediction probabilities near the decision threshold. These samples correspond to ambiguous instances where the model exhibits uncertainty. The observed behavior is consistent with clinically challenging cases, supporting the reliability and interpretability of the proposed approach.

Furthermore, borderline cases with prediction probabilities near the decision threshold were examined. As illustrated in Figure 4, these cases correspond to ambiguous instances where the model expresses uncertainty, which is consistent with clinically challenging scenarios.

## 6. Discussion and Conclusions

Chiari Malformation Type I (CM1) remains a challenging condition to evaluate radiologically because the anatomical complexity of the cranio-cervical junction cannot always be adequately described using a single morphometric parameter. In routine clinical practice, diagnosis often relies on manual measurements such as the degree of cerebellar tonsillar herniation. However, it is well recognized that this measurement alone does not consistently correlate with clinical symptoms or surgical outcomes. Previous studies have therefore suggested that additional parameters—including posterior fossa volume, brainstem morphology, and craniocervical angular relationships—may provide a more comprehensive assessment. Despite these efforts, there is still no widely accepted standardized framework that integrates these parameters into a consistent diagnostic workflow, which may lead to variability in interpretation and clinical decision-making.

In recent years, artificial intelligence and deep learning techniques have increasingly been applied to medical image analysis in order to identify complex anatomical patterns that may not be easily detected by manual evaluation. Convolutional neural networks (CNNs), in particular, have demonstrated strong performance across a variety of radiological classification tasks. However, fully end-to-end CNN models are often criticized for limited interpretability and reduced transparency in clinical contexts. In the present study, we therefore adopted a hybrid framework in which CNN architectures were used as feature extractors while classification was performed using conventional machine learning algorithms. This approach aims to retain the representational power of deep neural networks while allowing the final classification stage to be implemented using more interpretable and flexible models.

The findings of this study indicate that deep features extracted from sagittal T2-weighted MRI images contain highly discriminative information for the detection of CM1. In the hold-out test evaluation, ResNet-based feature representations achieved perfect classification metrics across all evaluated classifiers, whereas MobileNet-based features produced slightly lower accuracy and sensitivity values with a limited number of false-negative predictions but no false positives. This pattern suggests that most of the observed errors were related to missed positive cases rather than incorrect identification of healthy subjects. The consistent absence of false positives is particularly noteworthy from a clinical perspective, as it indicates that the model is unlikely to incorrectly label healthy individuals as having CM1.

The cross-validation analysis further supports the robustness of these findings. Across the five-fold cross-validation experiments, ResNet-based models consistently demonstrated extremely high and stable performance with accuracy, sensitivity, specificity, and AUC values equal to or very close to 1.00. MobileNetV2-based models also achieved strong performance but exhibited slightly lower mean accuracy values and somewhat greater variability across folds. Overall, MobileNetV2 accuracy values generally ranged between approximately 0.98 and 0.99, while AUC values remained very close to unity. These results suggest that both architectures provide strong discriminative capability; however, the ResNet-based representation appears to offer greater stability and robustness within the present dataset.

These observations are broadly consistent with previous studies that have applied deep learning approaches to CM1 imaging analysis. Lin et al. (2022) reported classification accuracies ranging from 97% to 100% using CNN models applied to T1-weighted sagittal MRI images [16]. Similarly, Tanaka et al. (2022) reported sensitivity and specificity values between 94% and 97% using ResNet-50 and VGG19 architectures [17]. Compared with these studies, the present work introduces several methodological differences. First, the analysis was performed using T2-weighted sagittal images rather than T1-weighted sequences. Second, both median and paramedian slices were included, which may allow the model to capture additional anatomical information related to the craniocervical junction. Third, images were obtained from two different MRI scanners, introducing a degree of acquisition variability that is often absent in single-scanner studies. Finally, instead of performing classification directly within CNN architectures, the extracted deep features were evaluated using multiple machine learning classifiers and ensemble methods.

Our results also compare favorably with studies based primarily on manually derived morphometric measurements. Mesin et al. (2022), for example, reported an accuracy of approximately 71% using morphometric parameters and clinical data in a pediatric cohort [18]. Similarly, Tetik et al. (2021) reported high diagnostic accuracy using posterior cranial fossa measurements [19]. While such approaches can provide valuable quantitative information, they rely on manual measurement procedures that may introduce observer-dependent variability. In contrast, the present study relies on automated feature extraction directly from MRI images, which may reduce subjectivity and improve reproducibility.

Another relevant study by El-Hajj et al. (2024) applied machine learning algorithms to a large dataset of 7106 patients in order to predict 30-day postoperative complications following Chiari decompression surgery, reporting an AUC of approximately 0.99 using a Random Forest model [21]. Although this study demonstrates the potential of machine learning for clinical prediction in Chiari-related conditions, its primary focus was postoperative outcome prediction rather than the imaging-based diagnosis of CM1. Furthermore, the analysis was not based on MRI image features. In contrast, the present study specifically addresses the diagnostic stage and focuses on learning discriminative features directly from MRI images.

Although ResNet-based models numerically outperformed MobileNetV2-based models in most experiments, statistical comparison tests did not reveal significant differences between the two architectures in the hold-out analysis. This finding likely reflects the extremely high performance achieved by both models and the very small number of discordant cases between them. When classification accuracy approaches a ceiling level, statistical comparison becomes less informative because only a limited number of misclassifications remain available for analysis. Nevertheless, the consistently higher mean performance and lower variance observed with ResNet-based models suggest that this architecture provides a more stable representation for CM1 classification.

The threshold sensitivity analysis provides additional insight into model stability. Across the evaluated threshold values, the ResNet-based model maintained perfect classification accuracy, whereas the MobileNet-based model exhibited a small increase in misclassifications as the decision threshold increased. This observation indicates that ResNet-derived feature representations remain robust under variations in decision threshold. In real-world clinical applications, such robustness may be important because classification thresholds may need to be adjusted according to disease prevalence, referral strategy, or clinical risk tolerance. From a clinical perspective, the proposed framework should be considered a decision-support system rather than a replacement for radiologist interpretation. In routine sagittal T2-weighted cervical MRI examinations, such a model could provide probability-based diagnostic support in borderline or ambiguous cases and potentially reduce inter-observer variability. Importantly, the results of this study demonstrate that strong diagnostic performance can be achieved using standard clinical imaging sequences without requiring specialized acquisition protocols.

The analysis of failure and borderline cases provides important insights into model reliability. While the ResNet-based model achieved perfect classification on the test set, such results should be interpreted with caution. This may reflect the relatively homogeneous nature of the dataset and the exclusion of complex clinical cases. Therefore, the observed performance should not be directly generalized to more heterogeneous real-world clinical populations without further external validation. In contrast, the MobileNet-based model exhibited a small number of misclassifications, which were associated with low-confidence predictions. This finding indicates that the model does not fail randomly, but instead expresses uncertainty in difficult or ambiguous cases. Such behavior is desirable for clinical decision-support systems, as it reduces the risk of overconfident errors. Overall, this behavior is consistent with clinical practice, where borderline cases are inherently difficult to classify even for experienced radiologists. To further validate that the classification task was not trivial, baseline models using DummyClassifier were evaluated. These models achieved accuracy close to chance level, whereas the proposed models demonstrated near-perfect performance. This substantial performance gap indicates that the observed results are attributable to the discriminative power of the extracted deep features rather than dataset simplicity.

Several limitations should be acknowledged. First, the study was conducted as a retrospective single-center analysis, which may limit the generalizability of the findings across institutions, scanners, and imaging protocols. Second, the reference standard was based on image labeling performed by a single experienced radiologist according to predefined diagnostic criteria. Although this approach reflects routine clinical practice, it may introduce some degree of observer-dependent bias, particularly in borderline cases. Third, the analysis relied exclusively on sagittal T2-weighted images and did not incorporate additional MRI sequences or clinical variables that could potentially improve classification performance. Finally, no external validation dataset was available; therefore, the reported results represent internal validation only. The lack of external validation represents an important limitation of this study. Therefore, the proposed approach should be considered as preliminary, and further evaluation on multi-center datasets is required before clinical deployment. Additionally, a per-scanner subgroup analysis could not be performed due to limited sample sizes within each subgroup, and this remains an important direction for future validation.

Future research should therefore focus on validating the proposed framework using larger multicenter datasets in order to evaluate its generalizability across different imaging protocols and patient populations. Prospective reader–AI comparison studies would also be valuable to determine whether such systems can improve diagnostic accuracy, reading efficiency, or diagnostic confidence in clinical practice. In addition, combining complementary features extracted from multiple CNN architectures may further improve classification robustness.

In conclusion, this study demonstrates that deep features extracted from sagittal T2-weighted MRI images can effectively capture morphological patterns associated with Chiari Malformation Type I. Among the evaluated approaches, ResNet-50–based feature representations provided the most stable and highest classification performance, while MobileNetV2-based models also achieved strong results with slightly lower stability. These findings suggest that hybrid frameworks combining CNN-based feature extraction with machine learning classifiers may represent a promising approach for developing clinically useful decision-support tools for CM1 detection.

## Figures and Tables

**Table 1:** Model default parameters.

Model	Default Hyperparameters
Support Vector Machine (SVM, RBF kernel)	C = 1.0, kernel = ‘rbf’, gamma = ‘scale’, degree = 3, coef0 = 0.0, probability = False (you enabled probability = True), shrinking = True, tol = 1 × 10^−3^, max_iter = −1
Logistic Regression (LR)	penalty = ‘l2’, solver = ‘lbfgs’, C = 1.0, max_iter = 100, fit_intercept = True, class_weight = None, tol = 1 × 10^−4^, multi_class = ‘auto’
Random Forest Classifier (RF)	n_estimators = 100, criterion = ‘gini’, max_depth = None, min_samples_split = 2, min_samples_leaf = 1, max_features = ‘sqrt’, bootstrap = True, n_jobs = None, random_state = None
XGBoost Classifier (XGB)	n_estimators = 100, learning_rate = 0.1, max_depth = 3, min_child_weight = 1, subsample = 1.0, colsample_bytree = 1.0, gamma = 0, reg_alpha = 0, reg_lambda = 1, objective = ‘binary:logistic’, eval_metric = ‘logloss’
VotingClassifier (Soft Voting)	voting = ‘soft’, weights = None, n_jobs = None, flatten_transform = True, verbose = False
VotingClassifier (Hard Voting)	voting = ‘hard’, weights = None, n_jobs = None, flatten_transform = True, verbose = False
Included Base Models (Default Settings)	SVM (kernel = ‘rbf’, C = 1.0, gamma = ‘scale’, probability = True); RandomForest (n_estimators = 100, criterion = ‘gini’); XGBoost (n_estimators = 100, learning_rate = 0.1, max_depth = 3, eval_metric = ‘logloss’)

**Table 2:** Voting classifier results.

Model	Feature	Accuracy	Balanced Acc	Sensitivity	Specificity	PPV	NPV	F1	AUC
SVM	ResNet	1.000	1.000	1.000	1.000	1.000	1.000	1.000	1.000
SVM	MobileNet	0.967	0.967	0.933	1.000	1.000	0.938	0.966	1.000
RF	ResNet	1.000	1.000	1.000	1.000	1.000	1.000	1.000	1.000
RF	MobileNet	0.983	0.983	0.967	1.000	1.000	0.968	0.983	1.000
LR	ResNet	1.000	1.000	1.000	1.000	1.000	1.000	1.000	1.000
LR	MobileNet	0.967	0.967	0.933	1.000	1.000	0.938	0.966	1.000
XGBoost	ResNet	1.000	1.000	1.000	1.000	1.000	1.000	1.000	1.000
XGBoost	MobileNet	0.967	0.967	0.933	1.000	1.000	0.938	0.966	1.000
VOTING_SOFT	ResNet	1.000	1.000	1.000	1.000	1.000	1.000	1.000	1.000
VOTING_SOFT	MobileNet	0.967	0.967	0.933	1.000	1.000	0.938	0.966	1.000
VOTING_HARD	ResNet	1.000	1.000	1.000	1.000	1.000	1.000	1.000	—
VOTING_HARD	MobileNet	0.967	0.967	0.933	1.000	1.000	0.938	0.966	—

**Table 3:** Final results N/A: Not Available.

Model	McNemar_*p*	AUC_ResNet	AUC_MobileNet	DeLong_*p*
SVM	0.479500122	1	1	1
RF	1	1	1	1
LR	0.479500122	1	1	1
XGBoost	0.479500122	1	1	1
VOTING_SOFT	0.479500122	1	1	1
VOTING_HARD	0.479500122	N/A	N/A	N/A

**Table 4:** Results of different thresholds.

Threshold	ResNet ACC	MobileNet ACC	a	b	c	d	McNemar *p*
0.1	1.000	1.000	60	0	0	0	—
0.2	1.000	1.000	60	0	0	0	—
0.3	1.000	1.000	60	0	0	0	—
0.4	1.000	0.983	59	1	0	0	1.000
0.5	1.000	0.967	58	2	0	0	0.480
0.6	1.000	0.967	58	2	0	0	0.480
0.7	1.000	0.967	58	2	0	0	0.480
0.8	1.000	0.967	58	2	0	0	0.480
0.9	1.000	0.933	56	4	0	0	0.134

**Table 5:** Paired 2 × 2 contingency table used for the McNemar test comparing ResNet and MobileNet classification outcomes.

	MobileNet Correct	MobileNet Incorrect
ResNet Correct	a	b
ResNet Incorrect	c	d

**Table 6:** K-fold = 5 cross-validation results.

Model	Feature	Accuracy (±SD)	Balanced Acc (±SD)	Sensitivity (±SD)	Specificity (±SD)	F1 (±SD)	AUC (±SD)
SVM	ResNet	1.000 ± 0.000	1.000 ± 0.000	1.000 ± ~0	1.000 ± ~0	1.000 ± 0.000	1.000 ± 0.000
SVM	MobileNet	0.990 ± 0.006	0.990 ± 0.006	0.986 ± 0.014	0.994 ± 0.008	0.990 ± 0.006	0.99976 ± 0.00027
RF	ResNet	1.000 ± 0.000	1.000 ± 0.000	1.000 ± ~0	1.000 ± ~0	1.000 ± 0.000	1.000 ± 0.000
RF	MobileNet	0.993 ± 0.005	0.993 ± 0.005	0.991 ± 0.013	0.994 ± 0.008	0.993 ± 0.005	0.99970 ± 0.00031
LR	ResNet	1.000 ± 0.000	1.000 ± 0.000	1.000 ± ~0	1.000 ± ~0	1.000 ± 0.000	1.000 ± 0.000
LR	MobileNet	0.993 ± 0.005	0.993 ± 0.005	0.991 ± 0.013	0.994 ± 0.008	0.993 ± 0.005	0.99984 ± 0.00017
XGBoost	ResNet	0.997 ± 0.006	0.997 ± 0.006	0.997 ± 0.006	0.997 ± 0.006	0.997 ± 0.006	0.997 ± 0.006
XGBoost	MobileNet	0.984 ± 0.009	0.984 ± 0.009	0.986 ± 0.014	0.983 ± 0.012	0.984 ± 0.009	0.99947 ± 0.00047
Voting Soft	ResNet	1.000 ± 0.000	1.000 ± 0.000	1.000 ± ~0	1.000 ± ~0	1.000 ± 0.000	1.000 ± 0.000
Voting Soft	MobileNet	0.990 ± 0.006	0.990 ± 0.006	0.986 ± 0.014	0.994 ± 0.008	0.990 ± 0.006	0.99963 ± 0.00039
Dummy Baseline	ResNet	0.50 ± 0.02	0.50 ± 0.0	0.999 ± 0.0	N/A	0.66 ± 0.002	N/A
Dummy Baseline	MobileNet	0.50 ± 0.02	0.50 ± 0.0	0.999 ± 0.0	N/A	0.66 ± 0.002	N/A

N/A: Not Available.

## Data Availability

Code and trained models are available from the corresponding author upon reasonable request; imaging data are not publicly available due to patient privacy and institutional restrictions.

## References

[1] Kahn E.N., Muraszko K.M., Maher C.O. (2015). Prevalence of Chiari I Malformation and Syringomyelia. Neurosurg. Clin. N. Am..

[2] Wang M., Hu Y., Zuo Y., Zhao P., Guo F. (2022). Diagnosis and treatment of Chiari malformation type 1 in children: Interpretation on international consensus document (2021). Chin. J. Neuromed..

[3] Hiremath S.B., Fitsiori A., Boto J., Torres C., Zakhari N., Dietemann J.-L., Meling T., Vargas M. (2020). The perplexity surrounding chiari malformations—Are we any wiser now?. Am. J. Neuroradiol..

[4] Milhorat T.H., Chou M.W., Trinidad E.M., Kula R.W., Mandell M., Wolpert C., Speer M.C. (1999). Chiari I Malformation Redefined: Clinical and Radiographic Findings for 364 Symptomatic Patients. Neurosurgery.

[5] Urbizu A., Martin B.A., Moncho D., Rovira A., Poca M.A., Sahuquillo J., Macaya A., Español M.I. (2018). Machine learning applied to neuroimaging for diagnosis of adult classic Chiari malformation: Role of the basion as a key morphometric indicator. J. Neurosurg..

[6] Eppelheimer M.S., Biswas D., Braun A.M., Houston J.R., Allen P.A., Bapuraj J.R., Labuda R., Loth D.M., Frim D., Loth F. (2019). Quantification of changes in brain morphology following posterior fossa decompression surgery in women treated for Chiari malformation type 1. Neuroradiology.

[7] Furtado S.V., Reddy K., Hegde A.S. (2009). Posterior fossa morphometry in symptomatic pediatric and adult Chiari I malformation. J. Clin. Neurosci..

[8] Urbizu A., Poca M.A., Vidal X., Rovira A., Sahuquillo J., Macaya A. (2014). MRI-based morphometric analysis of posterior cranial fossa in the diagnosis of chiari malformation type I. J. Neuroimaging.

[9] Meadows J., Kraut M., Guarnieri M., Haroun R.I., Carson B.S. (2000). Asymptomatic Chiari Type I malformations identified on magnetic resonance imaging. J. Neurosurg..

[10] Khan A.A., Bhatti S.N., Khan G., Ahmed E., Aurangzeb A., Ali A., Khan A., Afzal S. (2010). Clinical and radiological findings in Arnold Chiari malformation. J. Ayub Med. Coll. Abbottabad.

[11] Elster A.D., Chen M.Y. (1992). Chiari I malformations: Clinical and radiologic reappraisal. Radiology.

[12] De Oliveira Brito J.N.P., Dos Santos B.A., Nascimento I.F., Martins L.A., Tavares C.B. (2019). Basilar invagination associated with chiari malformation type I: A literature review. Clinics.

[13] Alperin N., Loftus J.R., Oliu C.J., Bagci A.M., Lee S.H., Ertl-Wagner B., Green B., Sekula R. (2014). Magnetic Resonance Imaging Measures of Posterior Cranial Fossa Morphology and Cerebrospinal Fluid Physiology in Chiari Malformation Type I. Neurosurgery.

[14] Gupta V.P., Xu Z., Greenberg J.K., Strahle J.M., Haller G., Meehan T., Roberts A., Limbrick D.D., Lu C. (2025). Using artificial intelligence to identify three presenting phenotypes of Chiari type-1 malformation and syringomyelia. Neurosurgery.

[15] Hosny A., Parmar C., Quackenbush J., Schwartz L.H., Aerts H.J.W.L. (2018). Artificial intelligence in radiology. Nat. Rev. Cancer.

[16] Lin W.-W., Liu T.-J., Dai W.-L., Wang Q.W., Hu X.-B., Gu Z.-W., Zhu Y.-J. (2022). Diagnostic performance evaluation of adult Chiari malformation type I based on convolutional neural networks. Eur. J. Radiol..

[17] Tanaka K.W., Russo C., Liu S., Stoodley M.A., Di Ieva A. (2022). Use of deep learning in the MRI diagnosis of Chiari malformation type I. Neuroradiology.

[18] Mesin L., Ponzio F., Carlino C.F., Lenge M., Noris A., Leo M.C., Sica M., McGreevy K., Fabrik E.L.A., Giordano F. (2022). A Machine Learning Approach to Support Treatment Identification for Chiari I Malformation. Appl. Sci..

[19] Tetik B., Mert Doğan G., Paşahan R., Durak M.A., Güldoğan E., Saraç K., Önal Ç., Yıldırım I.O. (2021). Multi-parameter-based radiological diagnosis of Chiari Malformation using Machine Learning Technology. Int. J. Clin. Pract..

[20] King V., Liu S., Russo C., Jayasekara M., Stoodley M., Di Leva A. (2024). Use of Artificial Intelligence in the Prediction of Chiari Malformation Type 1 Recurrence After Posterior Fossa Decompressive Surgery. Cureus.

[21] El-Hajj V.G., Ghaith A.K., Elmi-Terander A., Ahn E.S., Daniels D.J., Bydon M. (2024). Machine learning for enhanced prognostication: Predicting 30-day outcomes following posterior fossa decompression surgery for Chiari malformation type I in a pediatric cohort. J. Neurosurg. Pediatr..

